# CFD Modeling of Shear-Rate Distribution in a Bubble Column Bioreactor: Influence of Bubble Diameter and Lift Force

**DOI:** 10.1021/acsomega.5c06482

**Published:** 2025-11-20

**Authors:** Ana Carolina B. Silva, Mateus N. Esperança, Alberto C. Badino, Rodrigo Béttega

**Affiliations:** † Department of Chemical Engineering, Federal University of São Carlos, Rod Washington Luis, São Carlos, SP, Postal Code 13565-905, Brazil; ‡ Institute of Chemistry, University of São Paulo, Av. Professor Lineu Prestes, 748, São Paulo, SP, Postal Code 05508-000, Brazil; § Federal Institute of Education, Science, and Technology of São Paulo, Campus Capivari, Av. Dr. Ênio Pires de Camargo, 2971, Ribeirão, Capivari, SP, Postal Code 13365-010, Brazil

## Abstract

Shear rate (γ̇) is a critical parameter for evaluating the bioreactor performance. Average shear rate ( ${\mathrm{\gamma ̇}}_{av}$ ) traditionally characterizes the shear environment, allowing comparisons among different bioreactor designs based on microorganism morphology and viability. This study presents an innovative approach using computational fluid dynamics (CFD) to assess the influence of lift force on both maximum ${\mathrm{\gamma ̇}}_{\max}$ and average ( ${\mathrm{\gamma ̇}}_{av}$ ) shear rates in a bubble column bioreactor. By employing a two-fluid Eulerian model for gas–liquid multiphase simulations, the research explored a range of specific air flows ranging from 1 to 5 vvm, specific flow rate per volume of medium, which corresponds to the superficial velocities of 4.57 to 22.86 m/s and varied average bubble diameters. Notably, including the lift force amplified the impact of bubble diameter variations on local velocity gradients, significantly affecting shear rates. The findings suggest that ${\mathrm{\gamma ̇}}_{\max}$ may serve as a superior metric for comparing bioreactor performance, providing a robust framework for the optimization of biotechnological system design.

## Graphical abstract

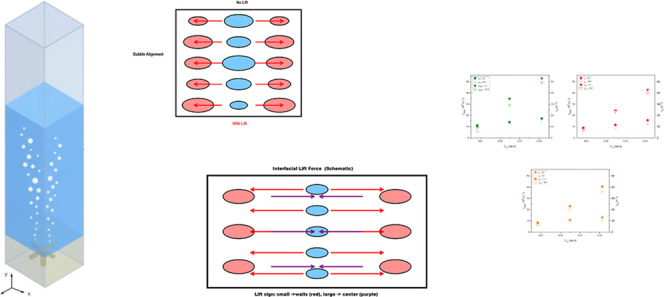

## Introduction

Growing cells in bioreactors are exposed to the hydrodynamic environment of the equipment, including shear stresses that can lead to irreparable morphological and physiological damage. Depending on the magnitude of these effects, losses in viability, cell rupture, and death can occur. As a result, the production of compounds of interest can be compromised, making the bioprocess in question unfeasible. To avoid damage to shear-sensitive cells, a bioreactor should provide moderate or low-shear environments, as long as it provides homogeneous heat and mass transfer distributions^1,2^ .

The shear rate (γ̇) is an important parameter in the design and operation of bioreactors. It helps estimate the magnitude of cellular damage in shear-sensitive biosystems and can be applied to correlate mass transfer and hydrodynamic parameters. The shear rate values vary with position, γ̇ = γ̇(*x*,*y*,*z*), reflecting local differences in fluid dynamics and mixing conditions. However, due to the complexity of determining the local shear rate, an average shear rate is usually considered, which is proportional to the superficial gas velocity.^3^ The fluid flow within bubble column bioreactors creates hydrodynamic stresses due to varying local velocities that generate a shear rate. Depending on cell size and structural complexity, whether referring to individual cells or larger cell aggregates, these shear forces can result in the disruption of single cells or the fragmentation of complex cellular structures.^4^

Interfacial forces play an important role in multiphase flows due to the momentum transfer across the interface, influencing the simulated dynamic patterns of fluids. The use of computational fluid dynamics (CFD) for modeling and simulation of multiphase flow has been widely applied to study these systems, as it allows for the prediction of momentum, heat, and mass transfers in bioreactors based on fundamental parameters.^5–7^ In multiphase flows, the lift force originates from the interaction between the local liquid velocity gradient and the bubble’s geometry.^8^ Under the conditions of a typical bubble column, where the liquid velocity profile near the walls is slower and, toward the center, faster, the lift force on smaller bubbles is positive, driving them toward the walls.^9^ This migration increases the local velocity gradient near the wall, which, in turn, raises the shear rate in those regions.

Hydrodynamics in bubble columns is chiefly governed by drag, while nondrag forces—especially lift (with diameter-dependent sign)—rearrange lateral bubble migration and the resulting mixing and shear patterns.^10,11^ Rectangular/square sections help isolate bubble diameter and lift effects by improving control of mesh symmetry and boundary conditions.^10^

In rectangular columns, combined experiments and CFD show that superficial gas velocity, sparger design, and bubble diameter shape circulation and high-shear regions; moreover, lift formulations (e.g., Tomiyama-type) affect velocity fields and phase distribution.^10,12,13^

For larger bubbles, the lift force reverses sign and becomes negative, which pushes the bubbles back into the reactor core, as demonstrated by Legendre and Magnaudet.^14^ Because of this, these bubbles are concentrated along the central axis, altering both the shear rate distribution and the overall mixing pattern in the fluid.^15^

The estimation of the shear rate in pneumatic bioreactors has been performed through theoretical analysis,^16^ intuitive analysis,^17–19^ analogical analysis,^3,20–23^ correlative analysis,^24^ and more recently applying computational fluid dynamics.^4,25–29^ These methods allow for a comparison between different bioreactor models. In conventional stirred and aerated tank bioreactors, the analysis of the maximum shear rate ( ${\mathrm{\gamma ̇}}_{\max}$ ) near to the sparger has led to a better understanding of the effects of shear in bioprocesses.^4,23,31^

In the study conducted by Jesus et al.^31^ comparing conventional bioreactors equipped with impellers, it was found that the shear level does not depend solely on the presence or absence of mechanical agitation but also on the operating conditions, such as agitation speed and gas flow rate. This behavior may be influenced by the parameter used to quantify the shear environment. In general, the shear conditions in different bioreactors were analyzed using the average shear rate ( ${\mathrm{\gamma ̇}}_{av}$ ). However, the use of the maximum shear rate ( ${\mathrm{\gamma ̇}}_{\max}$ ) represents a more assertive alternative for comparing bioreactors since it describes the worst condition to which microorganisms can be exposed inside the bioreactor.^4^

The values found for the average and maximum shear rates $({\mathrm{\gamma ̇}}_{av}$ and ${\mathrm{\gamma ̇}}_{\max})$ reported in the studies mentioned above are summarized in Table 1. Through the data, it is possible to observe that the ${\mathrm{\gamma ̇}}_{av}$ presented a wide range of values, regardless of the working volume, ranges of superficial gas velocities, or the nature of the fluid used.

**Table 1:** Studies Involving the Estimation of Average and Maximum Shear Rates ( ${\mathrm{\gamma ̇}}_{av}$ and ${\mathrm{\gamma ̇}}_{\max}$ ) in the Bubble Column Bioreactors^a^

references	volume (L)	fluids	${\mathrm{\gamma ̇}}_{\max}$ (s^–1^)	${\mathrm{\gamma ̇}}_{av}$ (s^–1^)
Nishikawa et al.^20^	32.0	W, MJS, CMCS, GS	-	0.1–500
Schumpe and Deckwer^24^	1.8/2.0/2.2	GS, CMCS, XGS, PAAS	-	56–560
Merchuk and Benzvi^14^	50	TW, GS, CMCS	-	68–611
Thomasi et al.^23^	5.0	W, GS, XGS	-	1000–8260 (XGS)
Esperança et al.^4^	5.0	W, GS, XGS	-	21.1–39.8
Esperança et al.^26^	5.0	W, GS	5834–24,397 (W) 5431–24,881 (GS)	11.6–13.0 (W) 11.0–15.1 (GS)

a CMCS: carboxymethylcellulose solution, GS: glycerol solution, MJS: millet jelly solution, PAAS: polyacrylamide solution, TW: tap water, W: water, and XGS: xanthan gum solution.

Table 1 raises concerns about the absolute accuracy of estimated average shear rates ( ${\mathrm{\gamma ̇}}_{av}$ ) obtained using existing literature methodologies. Because no direct experimental method exists to measure shear rate, accurately predicting ${\mathrm{\gamma ̇}}_{av}$ in pneumatic bioreactors remains elusive.^4^ Although previous studies have enhanced our understanding of how ${\mathrm{\gamma ̇}}_{av}$ affects shear-sensitive cells and microorganisms—thus enabling comparisons of shear conditions under various operating scenarios—determining the true average shear rate is still problematic. Consequently, relying solely on ${\mathrm{\gamma ̇}}_{av}$ to characterize shear conditions in bioreactors may be inadequate.

Using the maximum shear rate ( ${\mathrm{\gamma ̇}}_{\max}$ ) is more appropriate for comparing different bioreactor models because it reflects the worst-case conditions that microorganisms may encounter.^26^ Unlike the energy dissipation rate—which provides a bulk, averaged measure of energy transfer—the shear rate captures local hydrodynamic stresses directly linked to potential cell damage. In this context, ${\mathrm{\gamma ̇}}_{\max}$ represents a specific local value within the bioreactor. For instance, in stirred tank bioreactors, the highest shear occurs near the impeller,^25^ whereas in airlift bioreactors, the most severe shear conditions are found around the sparger holes in the bottom region.^27,28,32^

Computational fluid dynamics (CFD) can be used to estimate ${\mathrm{\gamma ̇}}_{\max}$ since it solves conservative equations (continuity and momentum), with the calculation of the flow fields and velocity profiles. This enables the estimation of local and average shear rates for different fluids and operating conditions. CFD has been widely used to evaluate the performance of pneumatic bioreactors by analyzing variables such as liquid velocity,^27,28,32–34^ global and local gas hold-up,^35–39^ and volumetric oxygen transfer coefficient.^27,35,40,41^ Nevertheless, few studies that use CFD to evaluate the shear conditions in pneumatic bioreactors are reported in the literature.^27,28,32,41,42^ Esperança et al.^4^ reported that CFD was a suitable tool to estimate the average shear rate ( ${\mathrm{\gamma ̇}}_{av}$ ) in pneumatic bioreactors, finding values for this parameter within an expected range of 11.0–27.3 s^–1^.

Since there is no agreement on the order of magnitude for average shear rates estimated using different methodologies and no consolidated procedure for determining this parameter, comparing these values with those obtained from CFD simulations is compromised. Therefore, an interesting approach would be to compare CFD-based shear rates to those obtained for a well-established simplified system. In this work, the influence of lift force and different average mean bubble diameters (4.0, 5.0, and 6.0 mm) on the maximum ( ${\mathrm{\gamma ̇}}_{\max}$ ) and average ( ${\mathrm{\gamma ̇}}_{av}$ ) shear rates in a square-section bubble column bioreactor were evaluated through CFD simulations and compared to the results obtained by Esperança et al.^26^

Recent studies in this field have mainly focused on the effect of the bubble diameter in cylindrical reactors. For example, Sun et al.^43^ investigated bubbles from 2 to 8 mm and showed that the maximum shear rate decreases nearly linearly with increasing diameter;^44^ demonstrated that larger bubbles yield lower gas hold-up and less intense shear peaks in diffuser-plate reactors;^45^ quantified how bubbles of 3, 5, and 7 mm affect velocity and shear-rate profiles in cylindrical columns. However, the study of a rectangular column with a captured lift-force effect across different bubble sizes is still poorly examined. In this sense, this work makes some novel contributions like a systematic mapping of both maximum and average shear rates for three bubble diameters (4, 5, and 6 mm) in a rectangular column with the gas phase modeled as a continuum in an Eulerian framework and the inclusion of the lift-force effect across those diameters, revealing the inversion of lift sign for the largest bubbles.

## Methodology

### Numerical Simulation

The 10 L square-section bubble column bioreactor^46^ was simulated using computational fluid dynamics (CFD). The simulations of the fluid dynamics were based on the previous studies of Rodriguez et al.^35^ and Esperança et al.^4^ To create the bubble column bioreactor computational geometry, the ANSYS Design Modeler was used (Figure 1).

**Figure 1:**
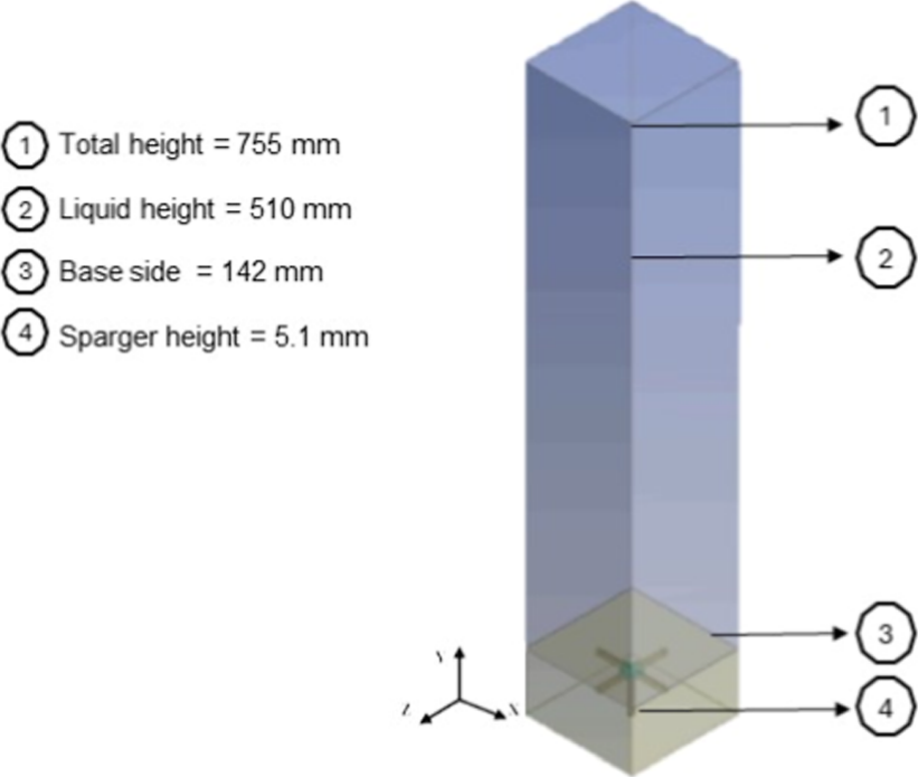
Computational geometry of the square-section bubble column.

ANSYS Meshing was used to generate the mesh grid (Figure 2a and b) and consisted of approximately 527,773 elements distributed in a structured hexahedral mesh for all the regions in the column, resulting in 100% hexahedral elements. The minimum and maximum node spacings were set at 0.45 and 4.0 mm, respectively, resulting in a 3.28 mm characteristic mesh size, defined as the cubic root of the domain volume divided by the total number of elements.^47^

**Figure 2:**
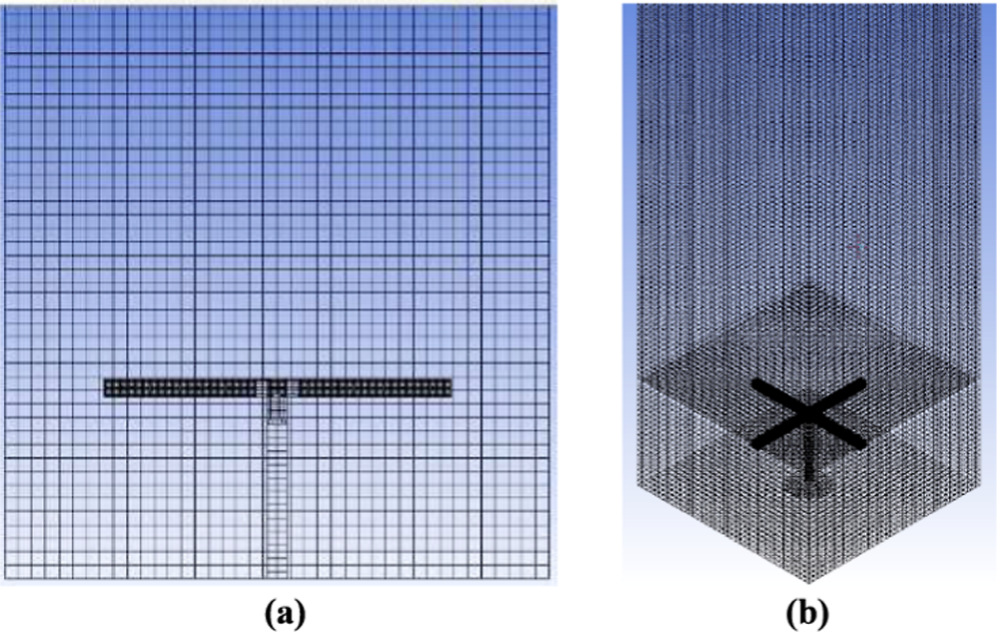
Computational grid details: (a) front view and (b) top view of the sparger area.

The mesh showed asymmetry values lower than 0.5 and orthogonal quality values higher than 0.8, indicating a high-quality mesh design. Additionally, the mesh elements presented aspect ratio values lower than 1.9, confirming the quality of the mesh used.

The grid-convergence study was performed with three meshes (769,338; 527,773; and 293,376 cells), using the global gas holdup (α̅_G_), the volumetric oxygen transfer coefficient (k_L_a), and the average shear rate ( ${\mathrm{\gamma ̇}}_{AV}$ ) as response variables. Only small percentage differences were observed between the finest and intermediate meshes, e.g., 3.00, 4.17, and 3.78, respectively, yielding an asymptotic solution ratio of 66.2% and low discretization errors. For the grid used in this study, 84,23% of the mesh elements showed equiangle and equisized skew values lower than 0.50, indicative of satisfactory mesh quality. In addition, 98.42% of the mesh elements presented aspect ratio values lower than 1.9, confirming good mesh quality.

The rectangular cross-section (0.142 × 0.755 m) allows the generation of a high-quality, structured hexahedral mesh in a Cartesian framework, which improves convergence and minimizes numerical diffusion in regions of strong velocity gradients. By using straight walls and right-angled corners, uniform cell sizes and simpler implementation of boundary conditions can be ensured, which makes it easier to isolate the effects of bubble diameter and lift force on shear distributions without the added complexity of curved geometry.

An Eulerian two-fluid model was used to describe the bioreactor hydrodynamics, considering the liquid as a continuous phase and the gas as dispersed bubbles. The diameters of the bubbles applied in the Eulerian gas phase varied in the simulations, ranging between 4.0 and 6.0 mm. This range was chosen based on previous studies from our group, which identified an average bubble diameter of approximately 5 mm, a finding supported by experimental data. The interphase momentum exchange forces considered in the simulations were the drag and lift forces, with the application of the Grace drag coefficient model,^48^ which considers different shapes of bubbles for the estimation of the drag coefficient. For the lift coefficient, the model of Tomiyama^49^ was considered, since it refers to air–water systems applying to deformable bubbles. The actions of the lift force in the bioreactor are shown in Figure 3.

**Figure 3:**
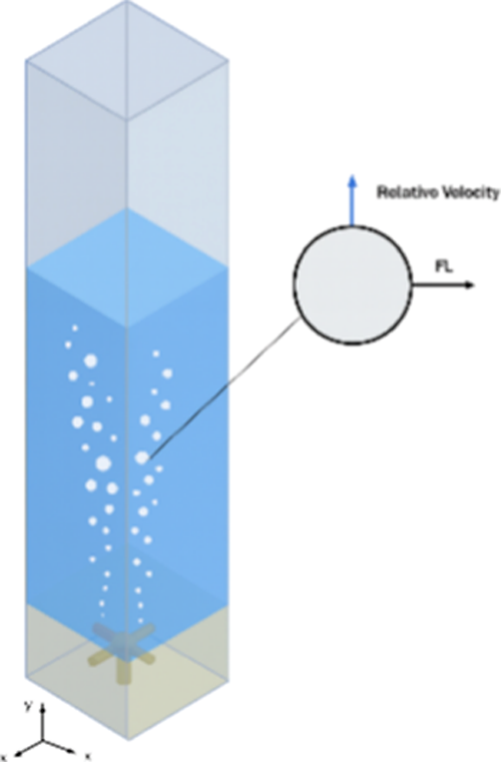
Interfacial lift force acting in the square-section bubble column: FL is the lift force.

It can be noticed that the sign of the lift force is size dependent, meaning the direction of the force acting on the bubbles changes with their diameter. Smaller bubbles typically experience an upward lift force, while larger bubbles can encounter a reversed force direction due to altered hydrodynamic interactions.^49^ This variation significantly influences bubble trajectories and the local shear rate distribution, which, in turn, impacts the overall mixing and performance in pneumatic bioreactors.

The turbulence was modeled by using the standard κ–ε model (Table 2). The transport equations for the turbulent kinetic energy (*k*) and its dissipation rate (ε) were described by eqs 1 and 2:

1 $$\frac{\partial }{\partial t}\left(\alpha_{i}\cdot \rho_{i}\cdot k_{i}\right)+\nabla \cdot \left(\alpha_{i}\left(\rho_{i}\cdot v_{i}\cdot k_{i}-\left(\mu_{i}+\frac{\mu_{t,i}}{\sigma_{k}}\right)\nabla k_{i}\right)\right)=\alpha_{i}\cdot \left(P_{i}-\rho_{i}\cdot \varepsilon_{i}\right)$$

2 $$\frac{\partial }{\partial t}\left(\alpha_{i}\cdot \rho_{i}\cdot \varepsilon_{i}\right)+\nabla \cdot \left(\alpha_{i}\cdot \rho_{i}\cdot v_{i}\cdot \varepsilon_{i}-\left(\mu_{i}+\frac{\mu_{t,i}}{\sigma_{\varepsilon }}\right)\nabla \varepsilon_{i}\right)=\alpha_{i}\cdot \frac{\varepsilon_{i}}{k_{i}}\left(C_{\varepsilon 1}\cdot P_{i}-C_{\varepsilon 2}\cdot \rho_{i}\cdot \varepsilon_{i}\right)$$

**Table 2:** Main Conditions and Models Used in the CFD Simulations

fluids	
water	continuous phase, ρ_L_ = 997 kg m^–3^, μ_L_ = 8.49.10^–4^ Pa s, σ = 0.072 N m^–1^
air	continuous phase, *d*_B_ = 4.0, 5.0, and 6.0 mm, ρ_G_ = 1.2 kg m^–3^
Boundary Conditions	
sparger holes	velocity component normal to boundary, gas volume fraction = 1.0
bioreactor top	prescribed outlet pressure (1 atm)
walls	nonslip condition for both phases
Turbulence	
liquid phase	dispersed phase Standard κ–ε model, *C*μ = 0.09, *C*ε_1_ = 1.44, *C*ε_2_ = 1.92, σk = 1, σε = 1.3
gas phase	dispersed phase Standard κ–ε model, *C*μ = 0.09, *C*ε_1_ = 1.44, *C*ε_2_ = 1.92, σk = 1, σε = 1.3
Interphase Momentum exchange force	
drag	Grace et al. (1976)
lift	Tomiyama (1998)
Solver Parameters	
solver	pressure based; Eulerian; implicit; two phases
velocity formulation	absolute
transient formulation	first-order implicit
pressure–velocity coupling scheme	phase-coupled simple
gradient discretization	least squares cell-based
momentum discretization	second-order upwind
volume fraction discretization	quick
turbulent kinetic energy	second-order implicit
turbulent dissipation rate	second-order implicit
residuals	RMS (root-mean-square) = 10^–4^
transient regime	25 iterations/time step, time step = 10^–4^ s, 50,000-time steps, simulated time = 5 s
under-relaxation factors	between 0.5 and 1.0

The gas injection boundary condition was defined as an inlet velocity at the sparger holes (volume fraction of air = 1), using values from 4.57 to 22.86 m s^–1^ for each hole, to give specific air flow rates ranging from 1.0 to 5.0 vvm. Although these velocities are locally high and resemble jetting conditions, they rapidly dissipate in the liquid bulk, resulting in bubble formation, as expected in gas–liquid systems. For the bioreactor walls, a no-slip condition was applied to both the liquid and gas phases to accurately capture the behavior at solid surfaces, assuming that the fluid velocity relative to the wall is zero. This common assumption in multiphase CFD models ensures that momentum exchange and boundary layer effects are properly represented, which is crucial with the influence of lift force on shear rates in pneumatic bioreactors. A pressure condition of 1 atm at the outlet boundary was specified (Table 2).

The governing equations were solved with ANSYS FLUENT 14.5 software and hardware consisting of an 8-core AMD Ryzen 7 3700x processor (3.59 GHz, 32 GB of RAM). The total simulation time was set to 15 s with a fixed 10^–4^ s time step. This simulated time value was chosen to be higher than the experimental liquid circulation time. This allows the liquid phase to complete at least one full circulation passage inside the bioreactor. The 15 s of simulated time was sufficient for pseudosteady-state behavior to be achieved for the independent variables, like air volume fraction, liquid velocities, and shear rate. Since the flow is highly transient, the reported shear rate values, both for the mean and maximum ( ${\mathrm{\gamma ̇}}_{av}$ and ${\mathrm{\gamma ̇}}_{\max}$ ), were evaluated using the volumetric averages over the entire computational domain and temporal averages in the pseudosteady interval of 10 to 15 s of simulated time. This procedure allows for the representation of the characteristic behavior of the bubble column bioreactor.

For the Eulerian phase, the air was considered as the dispersed phase with the bubble diameter varying from 4.0 to 6.0 mm. The convergence criterion used was RMS <10^–4^ (Table 2).

In this work, the second-order discretization scheme was chosen for the velocity formulation and for the turbulent kinetic energy and turbulent dissipation rate formulations. This choice was because more detailed assessments were evaluated and then a higher-order scheme was needed to avoid numerical diffusion. Further details concerning the computational grid, mathematical modeling, and numerical solution can be found in the work of Silva et al.^46^

A Courant–Friedrichs–Lewy (CFL) analysis was carried out to choose Δ*t*. By varying Δ*t* between 0.005 and 0.02 s, it was found that Δ*t* = 0.01 s maintains CFL <0.3 and converges within 500 iterations, balancing accuracy and computational cost. The simulated conditions were described as case studies and are presented in Table 3. To understand the nomenclature, the case study NLF4-3, for example, considers the following conditions: nonuse of lift force, d_B_ = 4.0 mm, and ϕ_air_ = 3.0 vvm, respectively.

**Table 3:** Nomenclature of Simulated Cases Using Computational Fluid Dynamics^a^

bubble diameter (mm)	cases	lift force	air flow (vvm)
4	LF4-1, LF4-3, and LF4-5	yes	1.0, 3.0, and 5.0
5	LF5-1, LF5-3, and LF5-5		
6	LF6-1, LF6-3, and LF6-5		
4	NLF4-1, NLF4-3, and NLF4-5	no	
5	NLF5-1, NLF5-3, and NLF5-5		
6	NLF6-1, NLF6-3, and NLF6-5		

a NLF: nonlift force and LF: with lift force.

## Estimation of the Analyzed Parameters

### Average Shear Rate ( ${\mathrm{\gamma ̇}}_{av}$ )

The values of the average shear rate ( ${\mathrm{\gamma ̇}}_{av}$ ) for bubble column bioreactors were estimated using the semiempirical correlation proposed by Pérez et al.^16^ which considers only the agitation as the input of pneumatic energy provided by the isothermal expansion of the gas entering the sparger. In this correlation, *P*_G_ refers to the power consumption, and *K* and *n* are the liquid rheological parameter consistency and flow indexes, respectively. In such cases, ${\mathrm{\gamma ̇}}_{av}$ depends exclusively on the superficial gas velocity and the rheological properties of the fluid, considering the experimental data of gas retention ( ${\mathrm{\alpha ̅}}_{G}$ ), according to eq 3.

3 $${\mathrm{\gamma ̇}}_{av}={\left(\frac{1}{K}\frac{P_{G}}{V}\right)}^{\left(1/n+1\right)}$$

The values of the average shear rate ( ${\mathrm{\gamma ̇}}_{av}$ ) were also obtained numerically, adopting the volume average procedure for the spatial distribution of the shear rate (γ̇) in the entire computational domain, using the Ansys CFD-Post tool. The values were compared with those obtained by the correlation and the results of Esperança et al.^4^

### Maximum Shear Rate ( ${\mathrm{\gamma ̇}}_{\max}$ )

The percentage difference between simulated data points was used to estimate the maximum shear rate ( ${\mathrm{\gamma ̇}}_{\max}$ ) near the sparger holes based on simulation, using the following equation

4 $$\Delta_{\%}\left(z\right)=\frac{{\mathrm{\gamma ̇}}_{\max,lift}\left(z\right)-{\mathrm{\gamma ̇}}_{\max,nolift}\left(z\right)}{{\mathrm{\gamma ̇}}_{\max,nolift}\left(z\right)}\times 100\%$$

which calculates the absolute difference between the two simulated values, divides it by the first simulated value, and then multiplies by 100 to express the difference as a percentage. This method allows us to determine the percentage difference between the two simulated data points.

Using the Ansys CFD-Post tool, fluid velocity profiles were generated in a small region adjacent to the sparger inlet—where shear effects are strongest—and values of ${\mathrm{\gamma ̇}}_{\max}$ were extracted from that zone. The model given by eq 5 was then fitted to these localized numerical values to estimate the proportionality constant *k*, as proposed by Esperança et al.^26^

5 $${\mathrm{\gamma ̇}}_{\max}=k\frac{4\cdot Q_{AR}}{N_{orif}\cdot \pi \cdot d_{orif}^{3}}$$

In Equation 5, *k* is a constant that depends on the pre-established conditions of the modeling, i.e., the bubble diameter variations, air flow (*Q*_AR_) rate, and the inclusion or not of the lift force; and *N*_orif_ and *d*_orif_ are the number of the hole and the diameter of the hole, respectively.

## Results and Discussion

### Average Shear Rate ( ${\mathrm{\gamma ̇}}_{av}$ )

Figure 4 summarizes the results of the average shear rate ( ${\mathrm{\gamma ̇}}_{av}$ ) with and without lift force, for the modeling considering diameters 4.0, 5.0, and 6.0 mm and airflow rates of 1.0, 3.0, and 5.0 vvm. The numerical results are compared to those obtained by the correlation proposed by Pérez et al.^16^ (eq 3) (to evaluate the order of magnitude of the parameter) and with results from Esperança et al.^4^

**Figure 4:**
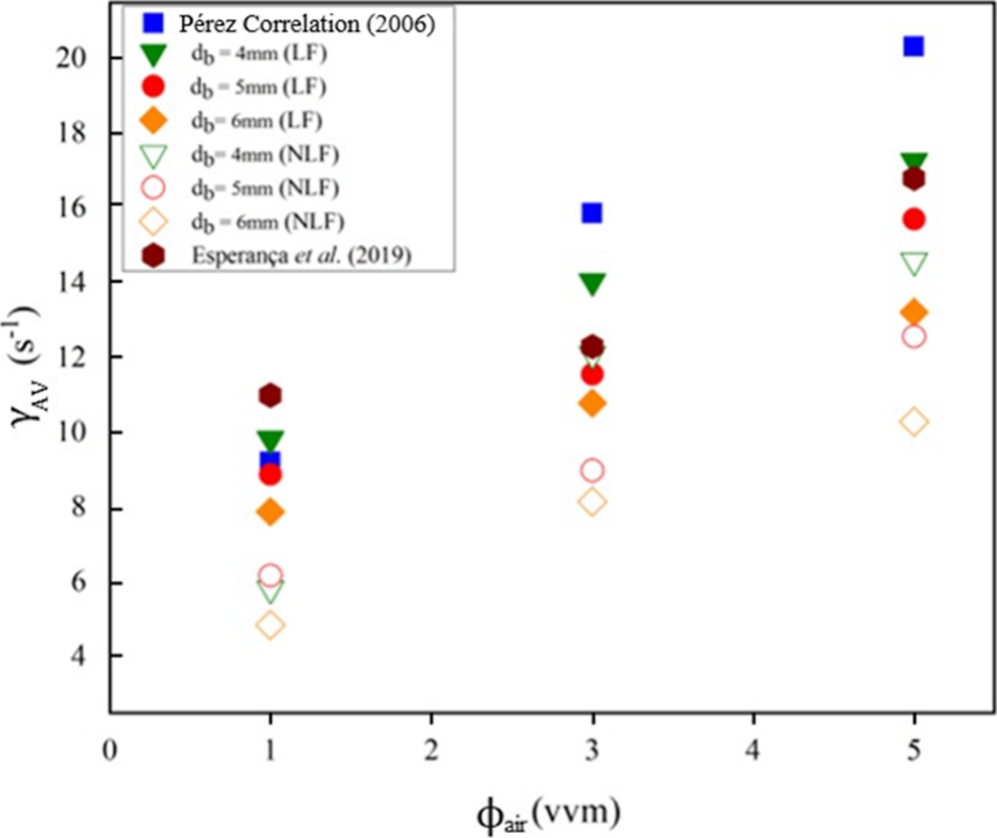
Average shear rate ( ${\mathrm{\gamma ̇}}_{av}$ ) as a function of the specific air flow rate (ϕ_air_) with and without lift force in the modeling (LF4-1 to LF6-5 and NLF4-1 to NLF6-1) obtained using eq 3.

The values of the average shear rate ( ${\mathrm{\gamma ̇}}_{av}$ ) obtained through computational fluid dynamics at a low air flow rate (1.0 vvm) exhibited a narrow range of variation for the cases NLF4-1, NLF5-1, and NLF6-1 (6.18, 5.83, and 4.85 s^–1^, respectively) and LF4-1, LF5-1, and LF6-1 (8.41, 7.72, and 6.88 s^–1^, respectively). Furthermore, the simulated values showed a good agreement with the data estimated by eq 3 and with the values obtained by Esperança et al.^4^ using CFD.

At the highest airflow rates (3.0 and 5.0 vvm), it was noted that the average shear rate values obtained from CFD simulations performed with and without incorporating the lift force were lower than the values estimated from the global gas holdup ( ${\mathrm{\alpha ̅}}_{G}$ ), using Pérez correlation, described in eq 3, for the studied diameters (4, 5, and 6 mm).

Figure 4 reveals that the average shear rate ( ${\mathrm{\gamma ̇}}_{av}$ ) exhibited lower values for larger bubble diameters. Still, these values were lower than those estimated by eq 3, which correlate ${\mathrm{\gamma ̇}}_{av}$ to the specific airflow rate, the power consumption, and gas holdup from experimental data. Despite the differences in the shear rate values obtained from CFD simulations, by the Pérez et al.^16^ correlation and the CFD simulation conducted by Esperança et al.,^4^ the general behavior and magnitude of the simulated ${\mathrm{\gamma ̇}}_{av}$ values were consistent with those they were compared against.

The authors evaluated the influence of airlift bioreactor geometry on the average shear rate using different Newtonian and non-Newtonian fluids, focusing solely on the drag force. The authors reported that for the water–air system, the average shear rates obtained by CFD simulation diverged from the values predicted by correlations using the liquid velocity profiles. However, the values presented the same order of magnitude and behavior with regard to the liquid velocity.

In this context, Figures 5 and 6 present the liquid-phase velocity contours at the final time step of the simulation—Figure 5 without the lift-force term and Figure 6 with it included. These snapshots are provided purely to visualize the flow field and illustrate how the lift force alters the liquid-velocity patterns, which are closely linked to local shear-rate distributions.

**Figure 5:**
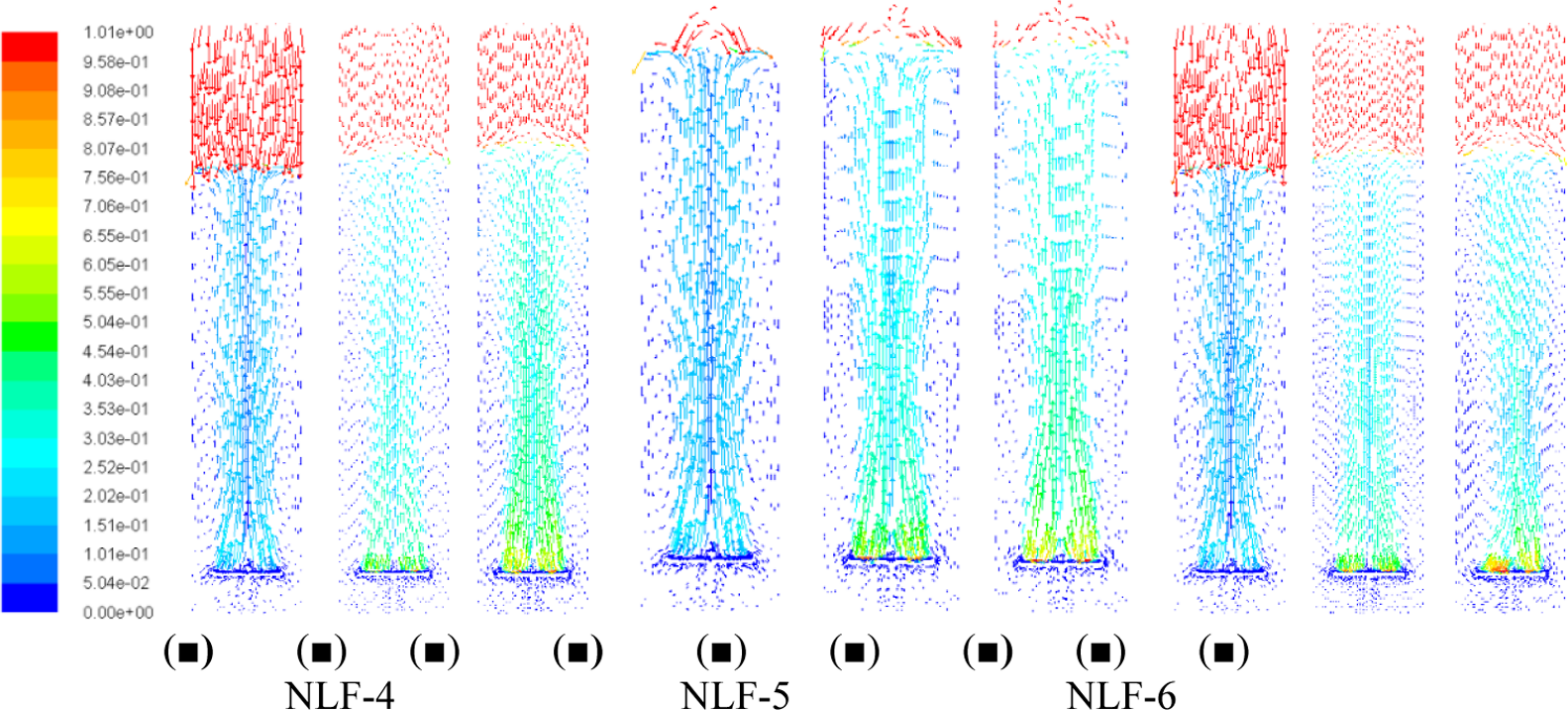
Contours of the liquid-phase velocity magnitude (m/s) in the bubble column without including the lift force and operating at the specific air flow rates of: (■) ϕ_air_ = 1.0 vvm, (■) ϕ_air_ = 3.0 vvm, and (■) ϕ_air_ = 5.0 vvm.

**Figure 6:**
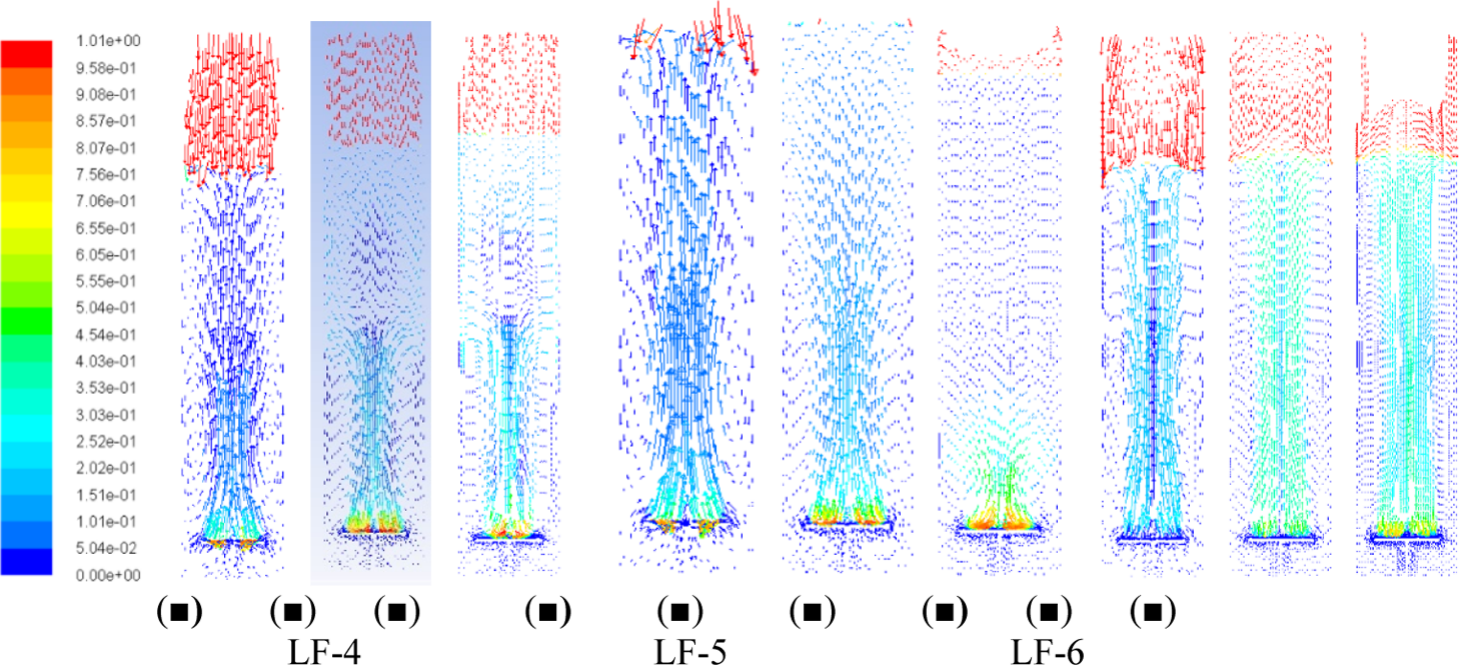
–Contours of the liquid-phase velocity magnitude (m/s) in the bubble column including the lift force and operating at the specific air flow rates of: (■) ϕair = 1.0 vvm, (■) ϕair = 3.0 vvm, and (■) ϕair = 5.0 vvm.

The axial profile of gas hold-up, ${\mathrm{\alpha ̅}}_{G}$ , shows that the largest difference in retention between the 4 mm and 5 mm cases occurs in the lower third of the column (z/H <0.3), immediately above the sparger. In that region, smaller bubbles (4 mm) experience stronger positive lift toward the walls, which prolongs their residence time in the liquid phase and elevates ${\mathrm{\alpha ̅}}_{G}$ . For the 5 mm bubbles, the lift is weaker, so they migrate less toward the walls, ascend more freely, and ${\mathrm{\alpha ̅}}_{G}$ peaks at a lower value and decay more rapidly with height. Figures 5 and 6 confirm that including lift increases liquid-phase velocities near the walls, promoting a more uniform bubble dispersion and validating the observed trends in the axial gas-hold-up profiles

### Maximum Shear Rate ( ${\mathrm{\gamma ̇}}_{\max}$ )

Maximum shear rate ( ${\mathrm{\gamma ̇}}_{\max}$ ) was evaluated using computational fluid dynamics (CFD) for models that considered, or did not consider, the lift force, across different bubble diameters. Equation 4 proposed by Esperança et al.^26^ was fitted to the simulated data and the values of the proportionality constant *k* were estimated for the different systems. Figures 7 and 8 illustrate the simulated values of ${\mathrm{\gamma ̇}}_{\max}$ and the fittings of eq 4 to the data, with and without the inclusion of the lift force, respectively.

**Figure 7:**
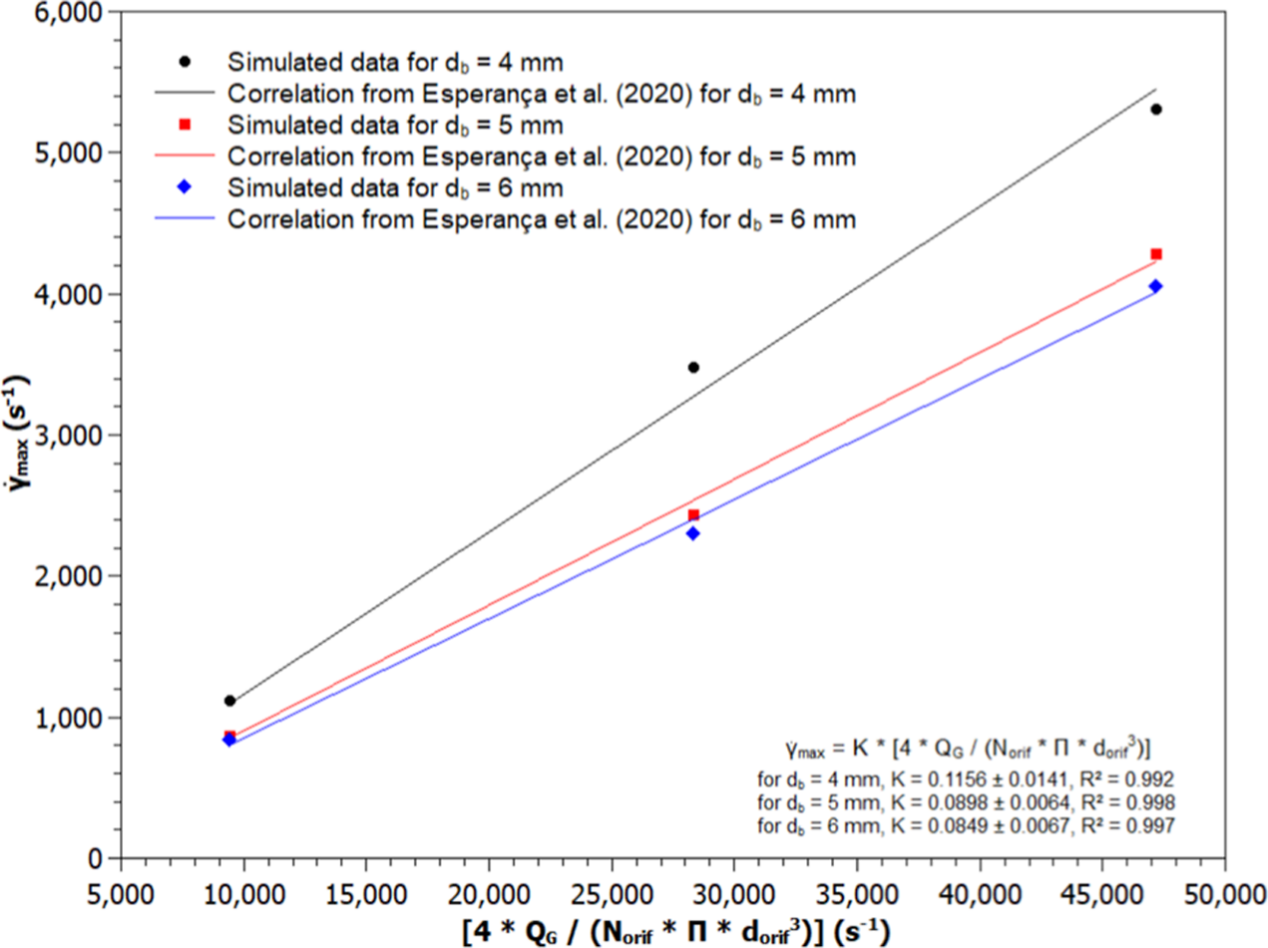
Maximum shear rate ( ${\mathrm{\gamma ̇}}_{\max}$ ) values obtained by CFD and the fitting of eq 5 (Esperança et al., 2020) in a bubble column bioreactor considering the lift force (LF4-1 to LF6-5): (a) d_b_ = 4.0 mm (●), (b) d_b_ = 5.0 mm (■), and (c) d_b_ = 6.0 mm (◆).

**Figure 8:**
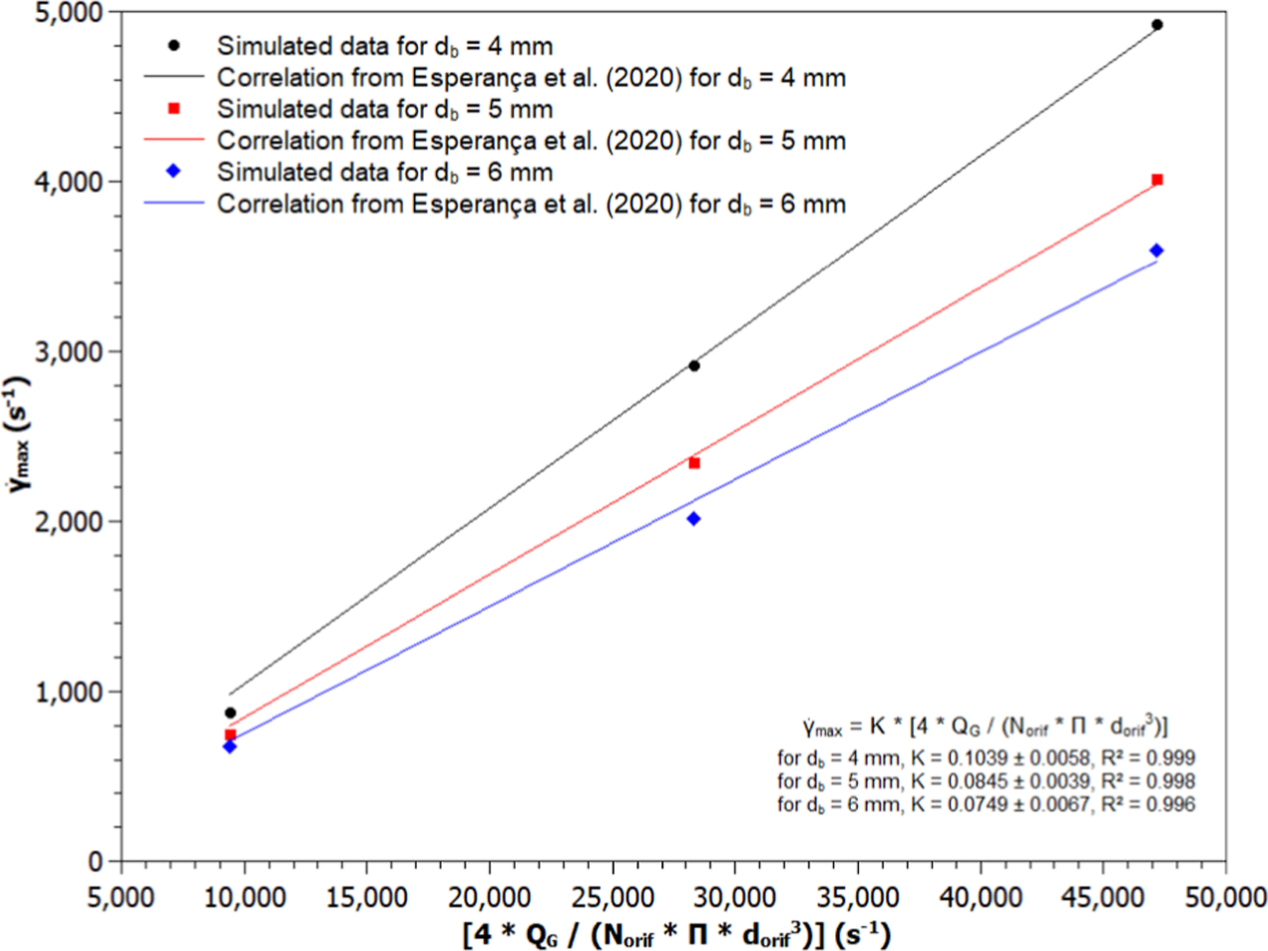
Maximum shear rate ( ${\mathrm{\gamma ̇}}_{\max}$ ) values obtained by CFD and the fitting of eq 5 (Esperança et al., 2020) in a bubble column bioreactor disregarding the lift force (NLF4-1 to NLF6-5): (a) d_b_ = 4.0 mm (●), (b) d_b_ = 5.0 mm (■), and (c) d_b_ = 6. mm (◆).

Since only three bubble-diameter cases were simulated, Figures 7 and 8 are interpreted qualitatively. In every case, the dominant shear-rate peak occurs right at the sparger exit (z/H ≈0), driven by jet impingement rather than lift. Further up the column, however, inclusion of lift produces a clear diameter-dependent effect: smaller bubbles migrate more strongly toward the walls, steepening local velocity gradients and increasing maximum shear rate ( ${\mathrm{\gamma ̇}}_{\max}$ ) in those regions compared to larger bubbles. Thus, the initial peak reflects impingement dynamics, while the downstream distribution of elevated shear highlights the contribution of the lift to bubble dispersion. Although three data points preclude robust statistical fitting, the pattern of a sparger-induced maximum followed by a lift-enhanced, size-dependent shear decline effectively demonstrates both mechanisms at work.

The proportionality constant *k* values with their respective standard deviations, for the cases that did not consider the lift force (NLF4 to NLF6), were equal to 0.1039 ± 0.0058, 0.0845 ± 0.0039, and 0.0749 ± 0.0067 for bubble diameters of 4.0, 5.0, and 6.0 mm, respectively. In the cases where lift force was considered (LF4 to LF6), the *k* values were equal to 0.1156 ± 0.0141, 0.0898 ± 0.0064, and 0.0849 ± 0.0067 for bubble diameters of 4.0, 5.0, and 6.0 mm, respectively. Analyzing these values, it is possible to observe that the insertion of the lift force in the simulations resulted in higher *k* values compared to the cases where this force was disregarded.

Figures 7 and 8 show that the maximum shear rate values $\left({\mathrm{\gamma ̇}}_{\max}\right)$ obtained in the simulations, with and without the lift force, were highest for the smallest diameter analyzed (4.0 mm) across all cases (NLF4-1 to NLF4-5 and LF4-1 to LF4-5). Furthermore, it is observed that when the lift force was not considered, *k* decreased as the mean bubble diameter increased. Such behavior may be linked to the fact that when the bubble diameter increases, the gas volumetric fraction might turn unstable,^50^ leading to higher velocity gradients. This behavior influences the maximum shear rate ( ${\mathrm{\gamma ̇}}_{\max}$ ) used in the fitting of eq 5.

Previous studies have shown that time-averaged shear rates above ≈2.5 s^–1^ induce membrane damage in human fibroblasts in stirred systems,^51^ while photobioreactor cultures of dinoflagellates suffer deleterious effects at average shear rates as low as ≈0.12 s^–1^. More robust mammalian lines such as CHO cells can tolerate transient shear peaks up to ≈700 s^–1^ before viability losses become significant.^52^ In the simulations present in this work, the maximum shear rates observed near the sparger lie within the critical range for many shear-sensitive cultures, underscoring the need to mitigate these hotspots in bioreactor design.

Adding any extra interfacial momentum term (e.g., lift) will necessarily cause the overall shear to increase, transferring more energy from the gas to the liquid, regardless of the motion of the gas itself. In the system presented in this study, the drag force

6 $$F_{D}=\frac{1}{2}C_{D}\rho_{L}A_{b}\left|u_{l}-u_{g}\right|\left(u_{l}-u_{g}\right)$$

is typically at least an order of magnitude larger than the lift force

7 $$F_{L}=\frac{1}{2}C_{L}\rho_{L}A_{b}\left(u_{l}-u_{g}\right)\omega_{l}$$

for the bubble sizes and shear rates studied, so drag remains the dominant interfacial momentum transfer. In the equations above, *F*_D_ is the drag force, *F*_L_ is the lift force, *C*_D_ and *C*_L_ are the drag and lift coefficients (respectively), ρ_L_ is the density of the liquid phase, *A*_b_ is the projected area of the bubble, ω_l_ is the vorticity of the liquid phase velocity field (s^–1^), and *u*_l_ and *u*_g_ are the velocities of the liquid and gas phases, respectively. Figure 9 compares the magnitudes of the drag and lift forces acting on single bubbles of 4, 5, and 6 mm in the rectangular column. As the bubble diameter increases, both forces decrease because larger bubbles experience lower relative velocity gradients and smaller interfacial area per unit volume.

**Figure 9:**
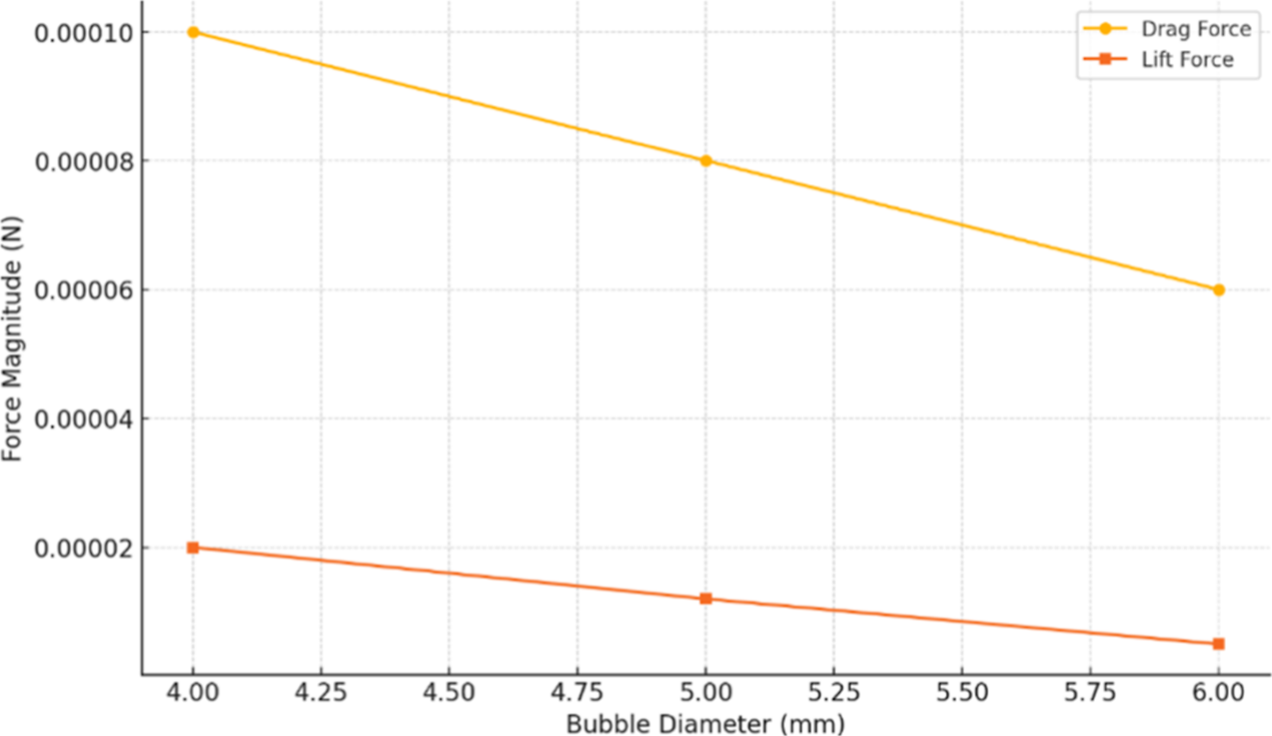
Drag and lift force magnitudes for the studied bubble diameters.

Importantly, drag remains roughly 4–5 times larger than lift across all sizes, confirming that drag is the dominant mechanism driving momentum transfer. However, the lift force is non-negligible—contributing up to ∼20% of the interfacial work for 4 mm bubbles—and its rapid decline with diameter explains why only the smallest bubbles show strong wall-ward migration and elevated shear near the walls in the simulations.

Figure 10 shows how the average shear rate ( ${\mathrm{\gamma ̇}}_{av}$ ) responds to increasing superficial gas velocity for bubble diameters of 4, 5, and 6 mm.

**Figure 10:**
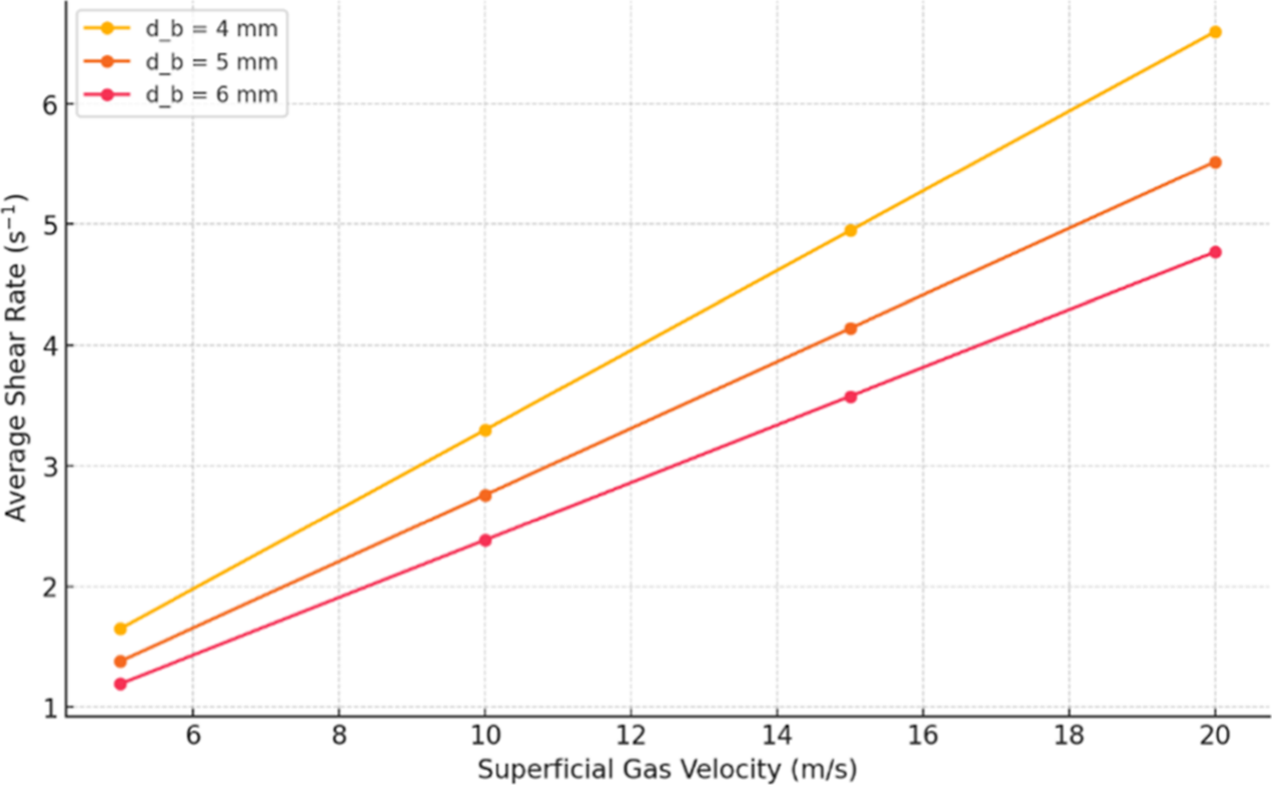
Sensitivity of ( ${\mathrm{\gamma ̇}}_{av}$ ) to gas velocity for the studied bubble diameters.

For all diameters, ( ${\mathrm{\gamma ̇}}_{av}$ ) increases nearly linearly with gas velocity, demonstrating that there is stronger net agitation at higher gas flows. The smallest bubbles (4 mm) produce a steeper slope—i.e., a greater increase in shear per unit gas velocity—because their larger interfacial area combined with positive lift toward the walls increases local velocity gradients. On the other hand, the largest bubbles (6 mm) produce a gentler slope, indicating less sensitivity of shear to gas flow. These trends corroborate our previous observations (Figures 5 and 6) that smaller bubbles generate higher shear rates.

In the simulations in which the lift force was considered, the average shear rate ( ${\mathrm{\gamma ̇}}_{av}$ ) exhibited higher values likely due to the influence of the lift force on the liquid velocity since ${\mathrm{\gamma ̇}}_{av}$ depends on this variable.

To understand the impact of varying bubble diameters and the consideration of the lift force on the maximum shear rate $\left({\mathrm{\gamma ̇}}_{\max}\right)$ , the percentage differences in the simulated data were calculated. To construct the data in Figure 11, first, paired simulations for each bubble diameter (4, 5, and 6 mm) at three gas velocities (4.57, 13.72, and 20.86 m/s) were run, including the lift force and once omitting it. For each case, the time- and volume-averaged maximum shear rate, ${\mathrm{\gamma ̇}}_{\max}$ , was evaluated over the pseudosteady interval (10–15 s) in thin axial slices. Then, the percentage difference was computed using eq 4 and using the 5 mm no-lift series (NLF-5) as the baseline reference. Plotting Δ_%_(*z*) for each diameter and velocity yields the bar chart of Figure 11.

**Figure 11:**
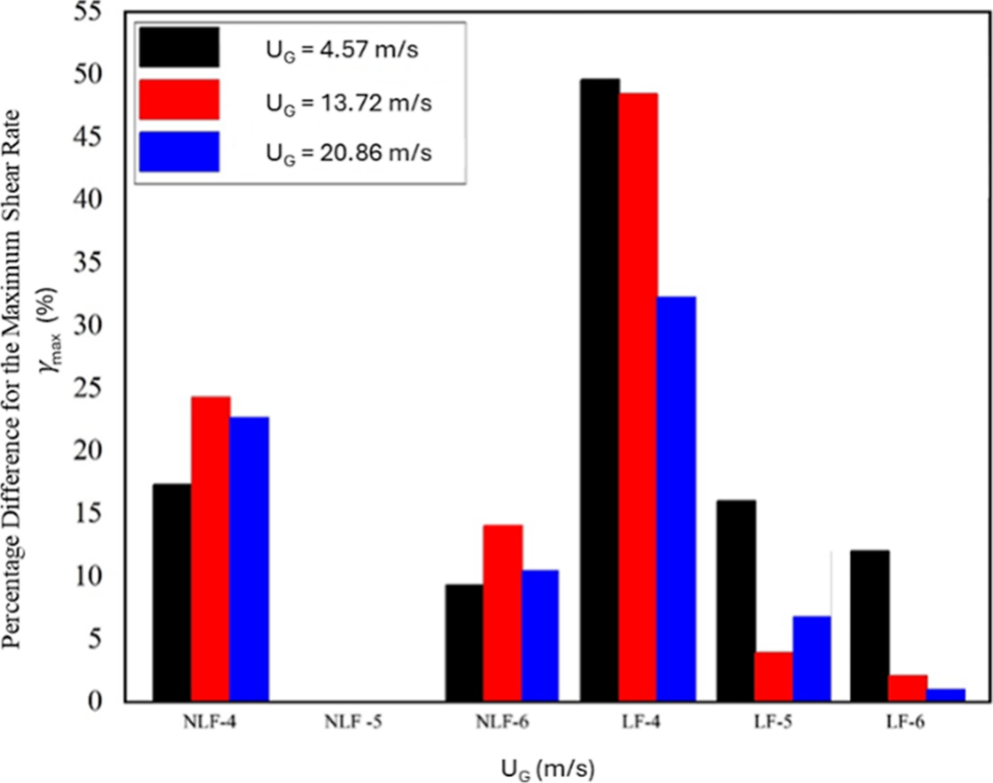
Percentage differences between simulated data points for the maximum shear rate ( ${\mathrm{\gamma ̇}}_{\max}$ ) considering the cases NFL5-1 to NLF5-5 as references.

This analysis is crucial because it isolates the sole contribution of lift to peak shear, independent of other model parameters. By expressing the change relative to a consistent reference case, we directly quantify how lift enhances or suppresses the maximum shear rate under varying flow and bubble-size conditions. Such insight guides bioreactor design by identifying the combinations of bubble size and gas velocity at which lift has the greatest impact on local shear stress, information that cannot be gleaned from absolute shear-rate values alone.

Figure 11 quantifies the percentage deviation of the time- and volume-averaged maximum shear rate ( ${\mathrm{\gamma ̇}}_{\max}$ ) for bubble diameters of 4, 5, and 6 mm at gas superficial velocities of 4.57, 13.72, and 20.86 m/s, using the 5 mm no-lift case (NLF-5) as the reference. Two key trends are apparent. First, inclusion of the lift force (LF series) dramatically amplifies ${\mathrm{\gamma ̇}}_{\max}$ for the smallest bubbles: 4 mm bubbles exhibit deviations of approximately 51% at 4.57 m/s, 49% at 13.72 m/s, and 32% at 20.86 m/s, reflecting enhanced wall-ward migration and steepened local velocity gradients. Second, the lift effect diminishes with increasing bubble size and gas velocity: in the LF-6 series, deviations drop to about 12%, 1.5%, and 2% at the same velocities, respectively. By contrast, the no-lift cases show much weaker diameter dependence, with 4 mm bubbles deviating by 17%–24% and 6 mm bubbles by only 10%–12%, independent of the flow rate. These findings demonstrate that lift-induced forces play a dominant role in elevating shear-rate peaks for small bubbles, particularly under low-to-moderate gas flow, but become progressively less significant for larger bubbles and higher velocities.

Esperança et al.^26^ performed a linear regression of 40 values of maximum shear rate for a constant diameter of 5.0 mm at specific air flow rates ranging from 1.0 to 5.0 vvm, considering only the drag force in concentric tube airlift bioreactors with working volumes of 5 and 10 L. The authors achieved a *k* value of 0.477 ± 0.008, and the values derived from eq 5’s correlation exhibited favorable alignment with those obtained via CFD. When compared to the simulated outcomes of the authors, for 10 L airlift split and concentric duct bioreactors employing spargers with 96 and 180 holes, this study observed a similar order of magnitude for the parameter *k* when applying the same type of sparger (crosshead). Results obtained by Esperança et al.^26^ for ${\mathrm{\gamma ̇}}_{\max}$ for the sparger with fewer holes were higher, as it resulted in higher values of gas injection velocity.

In general, the shear conditions in different bioreactors were analyzed using the average shear rate ( ${\mathrm{\gamma ̇}}_{av}$ ). However, the use of the maximum shear rate ( ${\mathrm{\gamma ̇}}_{\max}$ ) represents a more accurate alternative for comparing bioreactors, as it describes the worst condition to which a microorganism can be exposed inside a bioreactor.^26^ Thus, to demonstrate the influence of the lift force on the shear rate, a comparison between the maximum ( ${\mathrm{\gamma ̇}}_{\max}$ ) and average ( ${\mathrm{\gamma ̇}}_{av}$ ) shear rates in the bubble column bioreactor was made with the values obtained by CFD simulations, as shown in Figure 12(a), (b), and (c).

**Figure 12:**
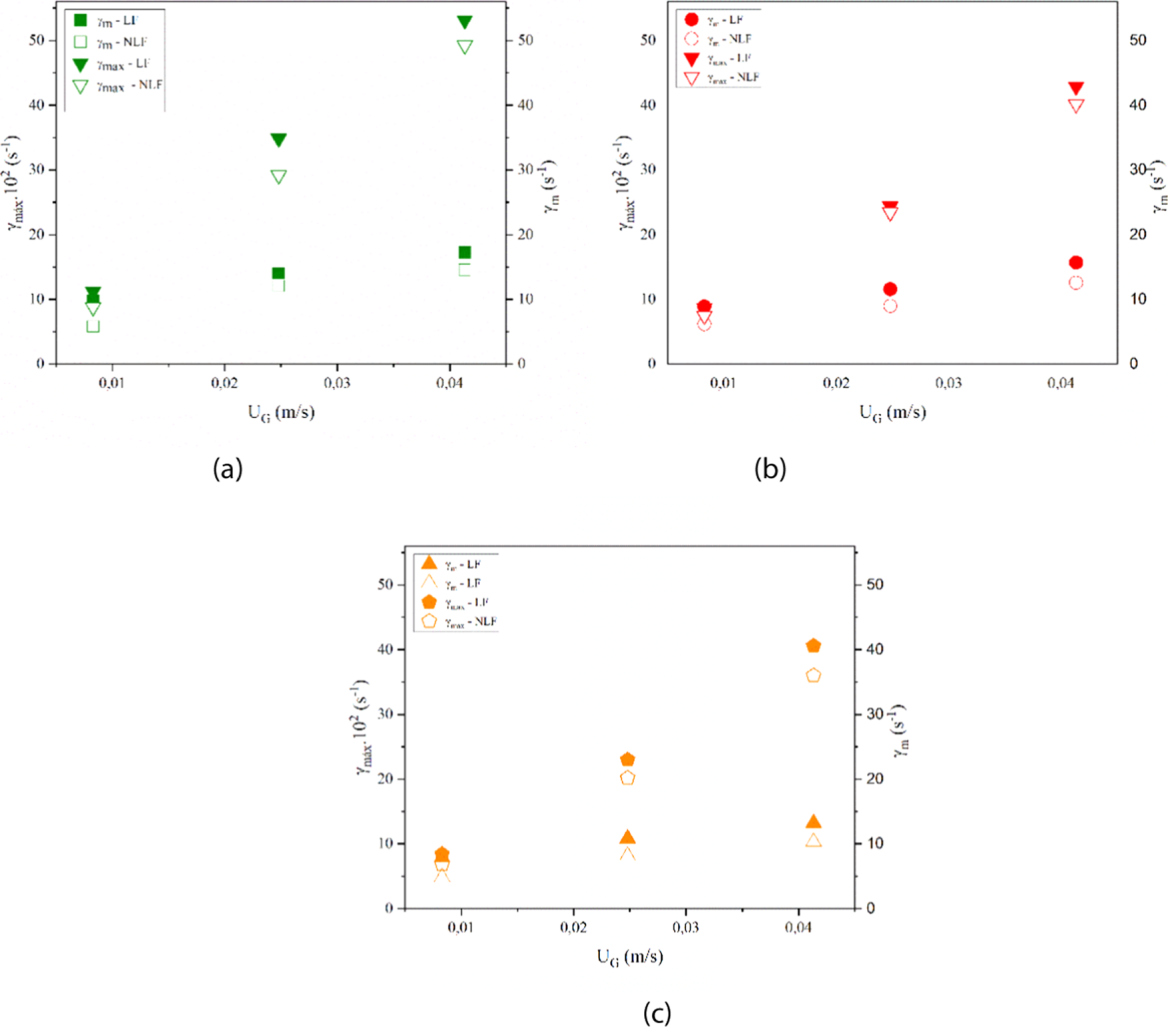
Comparison of the average ( ${\mathrm{\gamma ̇}}_{av}$ ) and maximum ( ${\mathrm{\gamma ̇}}_{\max}$ ) shear rates in the bubble column bioreactor, with and without the consideration of the lift force for: (a) *d*_B_ = 4.0 mm, (b) *d*_B_ = 5.0 mm, and (c) *d*_B_ = 6.0 mm.

Values of the average shear rate ( ${\mathrm{\gamma ̇}}_{av}$ ) considering the three specific air flow rates and the three bubble diameters used in this study ranged from 7.89 to 17.25 s^–1^ when the lift force was considered in the mathematical model (LF4-1 to LF6-5) and from 4.85 to 14.59 s^–1^ for cases in which this force was not considered (NLF4-1 to NLF6-5). However, the maximum shear rate values ( ${\mathrm{\gamma ̇}}_{\max}$ ) were in the range of 840 to 5312 s^–1^ for cases LF4-1 to LF6-5 and from 680 to 4928 s^–1^ for NLF4-1 to NLF6-5. Through this comparison, it is possible to observe that in the range of variation of the superficial gas velocity studied, the cases in which the lift force was considered showed higher average and maximum shear rate values.

It is known that the lift force is related to the lateral movement of the dispersed phase, in this case, the bubbles, acting laterally in ascending flows, where there are gradients in the liquid velocity. The greater this gradient, the greater the formation of eddies, favoring the exchange of mechanical energy between these vortices, making the system more turbulent, especially in the region of air injection. In this way, the consideration of the lift force helps to increase the values of the maximum shear rate, as it favors a greater dispersion of the gas phase in this specific area of the reactor.

Corroborating with the study by Esperança et al.,^4^ the results found in the present work showed that the maximum shear rate is a more suitable parameter to be used in the evaluation of the performance of the bioreactor than the average shear rate, due to the difference in the order of magnitude of the two quantities. It can be stated that there is a nonuniform spatial distribution of the average shear rate inside the equipment. In addition, operating conditions, such as the bubble diameter and liquid velocity, have been shown to play a key role in determining the shear environment.

## Conclusion

Computational fluid dynamics (CFD) was used to evaluate the influence of the interfacial lift force on the average ( ${\mathrm{\gamma ̇}}_{av}$ ) and maximum ( ${\mathrm{\gamma ̇}}_{\max}$ ) shear rates in a bubble column bioreactor for three different bubble diameters (4.0, 5.0, and 6.0 mm). At the same specific air flow rate, using the lift force, the smaller bubble diameter provided higher ${\mathrm{\gamma ̇}}_{av}$ and ${\mathrm{\gamma ̇}}_{\max}$ values compared with the diameters of 5.0 and 6.0 mm, when only the drag force was used. The average shear rate ( ${\mathrm{\gamma ̇}}_{av}$ ) presented a significant difference compared to the maximum shear rate ( ${\mathrm{\gamma ̇}}_{\max}$ ) of an order of magnitude, due to a nonuniform spatial distribution.

It was observed that the variation in the mean bubble diameter in the range evaluated in this study showed greater influence on the values of ${\mathrm{\gamma ̇}}_{\max}$ when the lift force was considered. This occurs because the maximum shear rate ( ${\mathrm{\gamma ̇}}_{\max}$ ) depends on the local velocity gradient, which is directly influenced by the lift force. Therefore, ${\mathrm{\gamma ̇}}_{\max}$ seems to be a more suitable parameter for describing the shear environment on the bubble column bioreactor, considering the lift force on the mathematical model.

## List of Symbols

C_ε1_: κ–ϵ turbulence model constant
C_ε2_: κ–ϵ turbulence model constant
C_μ_: κ–ϵ turbulence model constant
C_μp_: κ–ϵ turbulence model constant
*d*_b_: bubble diameter mm
*d*_hole_: hole diameter m
*N*_hole_: number of sparger holes
*Q*_G_: volumetric gas flow rate m^3^ s^–1^
*k*: consistency index Pa s^n^
κ: turbulent kinetic energy J kg^–1^

## Greek Letters

ε: turbulent kinetic energy dissipation rate J kg^–1^
ϕ_air_: specific air flow rate vvm
γ̇: shear rate s^–1^
${\mathrm{\gamma ̇}}_{av}$: average shear rate s^–1^
${\mathrm{\gamma ̇}}_{\max}$: maximum shear rate s^–1^
μ_*i*_: dynamic viscosity of phase “i” Pa s
ρ_*i*_: density of phase “i” kg m^–3^
σ_*k*_: κ–ε turbulence model constant for κ equation
σ_ε_: κ–ε turbulence model constant for ε equation
